# Exosome Release and Low pH Belong to a Framework of Resistance of Human Melanoma Cells to Cisplatin

**DOI:** 10.1371/journal.pone.0088193

**Published:** 2014-02-06

**Authors:** Cristina Federici, Francesco Petrucci, Stefano Caimi, Albino Cesolini, Mariantonia Logozzi, Martina Borghi, Sonia D'Ilio, Luana Lugini, Nicola Violante, Tommaso Azzarito, Costanza Majorani, Daria Brambilla, Stefano Fais

**Affiliations:** 1 Department of Therapeutic Research and Medicine Evaluation, National Institute of Health, Rome, Italy; 2 Department of Environment and Primary Prevention, National Institute of Health, Rome, Italy; 3 Department of Ematology, Oncology and Molecular Biology, National Institute of Health, Rome, Italy; 4 National Centre for Chemicals Substances, National Institute of Health, Rome, Italy,; Columbia University, United States of America

## Abstract

Intrinsic resistance to cytotoxic drugs has been a main issue in cancer therapy for decades. Microenvironmental acidity is a simple while highly efficient mechanism of chemoresistance, exploited through impairment of drug delivery. The latter is achieved by extracellular protonation and/or sequestration into acidic vesicles. This study investigates the importance of extracellular acidosis and nanovesicle (exosome) release in the resistance of human tumour cell to cisplatin (CisPt); in parallel to proton pump inhibitors (PPI) ability of interfering with these tumour cell features. The results showed that CisPt uptake by human tumour cells was markedly impaired by low pH conditions. Moreover, exosomes purified from supernatants of these cell cultures contained various amounts of CisPt, which correlated to the pH conditions of the culture medium. HPLC-Q-ICP-MS analysis revealed that exosome purified from tumour cell culture supernatants contained CisPt in its native form. PPI pre-treatment increased cellular uptake of CisPt, as compared to untreated cells, in an acidic-depend manner. Furthermore, it induced a clear inhibition of exosome release by tumour cells. Human tumours obtained from xenografts pretreated with PPI contained more CisPt as compared to tumours from xenografts treated with CisPt alone. Further analysis showed that *in vivo* PPI treatment induced a clear reduction in the plasmatic levels of tumour-derived exosomes which also contained lower level of CisPt. Altogether, these findings point to the identification of a double mechanism that human malignant melanoma use in resisting to a dreadful cellular poison such as cisplatin. This framework of resistance includes both low pH-dependent extracellular sequestration and an exosome-mediated elimination. Both mechanisms are markedly impaired by proton pump inhibition, leading to an increased CisPt-dependent cytotoxicity.

## Introduction

Malignant melanoma is among the most chemoresistant tumours. Commonly used anticancer drugs do not substantially alter the prognosis of the progressive disease. Single agent or combined chemotherapies result in poor benefits for patients with malignant melanoma [1], [2]. Standard treatment for metastatic melanoma based on the alkylating agent dacarbazine, frequently leads to poor outcomes [3], while combinations of chemotherapeutics have shown only marginally higher response rates, paying the price of systemic toxicity [4]. Such unsatisfactory treatments highlight the urgency of implementing treatment strategies for malignant melanoma with novel, more effective and possibly less toxic approaches. Despite some mechanisms of tumour resistance to a variety of cytotoxic drugs have been proposed in pre-clinical studies [5] the former do not seem to have a clear role in tumour patients and this appears even more evident for tumours that are non-responsive rather than resistant to chemotherapy, such as melanoma. Cisplatin (CisPt) is an alkylating agent that binds to DNA bases causing crosslinks and breaks in DNA strands; interfering with DNA replication [6]. An impaired uptake of CisPt appears to represent the most consistently identified feature of cells with resistance to this drug, both *in vitro* and *in vivo* [7], [8], as compared to other proposed mechanisms [9], [10]. The mechanism by which CisPt enters into the cells is unknown, but earlier evidence suggested that CisPt enters relatively slowly as compared to most anticancer drugs, whereas in turn CisPt efflux occurs rapidly [11].

One of the most important “entry regulators” of the vast majority of chemical drugs into the cells is a pH gradient between the extracellular and the intracellular compartments. In fact, a well known mechanism of chemoresistance is a reversed pH gradient that represents a hallmark of malignant tumours, with the development of an acidic extracellular pH (pHe) and an alkaline pH of the cytosolic compartment (pHi) of tumour cells [12], [13], [14]. The so called “Warburg Effect” features the triggering mechanism of extracellular acidity, caused by extracellular accumulation of lactate. However, this extracellular acidity conceivably selects cells with upregulated proton pumps activity that on one hand increases the acidity of the extracellular spaces and internal vesicles while on the other hand may lead to the alkalinization of the cytosolic compartment, as it occurs in drug-resistant cell lines [15], [16]. Several reports propose a role for acidic vesicles in resistance to cytotoxic drugs, through both the sequestration and neutralization of low alkaline drugs into the lumen of acidic organelles and the possible elimination of drugs from the cell through a vesicle-mediated secretory pathway [17], [18], [19].

The vacuolar ATPase (V-ATPase) is a proton pump responsible for acidification of lysosomes and regulation of vesicular traffic. In cancer cells, V-ATPase is involved in regulation of pHi and its expression and subcellular localization is associated to both metastatic capacity and multidrug-resistance [20], [21], [22]. Over the last 30 years, a class of H^+^-K^+^-ATPases (with many similarities with V-ATPase) inhibitors has been commonly employed as an antiacidic drug in the treatment of peptic diseases. The former are known as proton pump inhibitors (PPI) and include 6 molecules, all belonging to the same family. PPI treatment of both human tumour cell lines and tumours clearly induce cancer sensitivity for a variety of chemotherapeutics [23], [24]. This effect is consistent with an inhibition of both release and trafficking of acidic vesicles in human tumour cells [25]. Also CisPt may undergo sequestration into lysosomes and vesicles belonging to the secretory pathway [26], [27]. In human ovarian carcinoma cells, lysosomes and plasma membrane proteins are involved in CisPt efflux which originates from the trans-Golgi network and are often routed to multivesicular bodies, being subsequently destroyed in lysosomes or secreted into the extracellular environment via exosomes [28]. Exosomes are nanovesicles of endocytic origin, released by a variety of both normal and tumour cells. Exosomes have pleiotropic biological functions, including modulation of immune response, antigen presentation, intercellular communication and the intercellular transfer of RNA and proteins [29], [30]. We have recently shown that low pHe induces an increased release of exosomes by human melanoma cells and counteracting the low pH with either buffering of the tumour cell milieu or PPI treatment markedly reduced the exosome release from cancer cells [31].

This study was aimed at investigating the role of both extracellular acidosis and exosome release in resistance of melanoma cells to CisPt. Moreover, we evaluated the ability of PPI in restoring sensitivity of melanoma cells to CisPt, in both *in vitro* and *in vivo* experiments.

## Materials and Methods

### Cell lines

The cell lines MCF7 (human breast cancer, ATCC), Me30966 and Me501 (human metastatic melanoma), and SW480 (human colon carcinoma) supplied by Fondazione IRCCS Istituto Nazionale dei Tumouri, Milan, Italy [23], [31], were cultured in RPMI 1640 (Gibco Laboratories, Grand Island, NY, USA) supplemented with antibiotics (Sigma-Aldrich, St. Louis, MO) and 10% fetal bovine serum (FBS, Gibco) in humidified 5% CO_2_ and 95% air atmosphere. Human PBMC (Peripheral Blood Mononuclear Cells) were isolated from buffy coats by Ficoll-Histopaque 1077 gradient (Sigma-Aldrich). Buffy coats were provided by Centro Trasfusionale Universitario Azienda Policlinico Umberto I in Rome, Italy (the study was approved by the ethical committee of Istituto Superiore di Sanità, Rome, Italy, and donors gave written-informed consent to participate). Unbuffered culture medium (UNB) was prepared without sodium bicarbonate. Different pH mediums were controlled by a pH meter (Metrohm AG, mod. 691, Herisau, Switzerland). Experiments were performed in buffered medium (pH 7.4), unbuffered medium (UNB w/o sodium bicarbonate, initial pH 7.2) or buffered acidic medium (pH 5.0 or 6.0). The cell lines were negative for mycoplasma contamination, as routinely tested by modified nested polymerase chain reaction (VenorGeM Kit, Minerva biolabs, Germany).

### Drugs and reagents

PPI (Lansoprazole; Astra-Zeneca, Mölndal, Sweden) was resuspended in DMSO immediately before use. In combination treatment experiments, cells were pretreated for 24 hours with PPI and then treated for additional 6 hours with 2 µM Cisplatin (Teva Italia, Milan, Italy).

For the separation of the chemical forms of CisPt the following reagents were used: trifluoromethanesulfonic acid (triflic) (Sigma-Aldrich), methanol of chromatography grade (Lab Scan, Analytical Sciences, Dublin, Ireland), sodium dodecyl sulphate (SDS, Scientific Supplies, Auckland, NZ), sterile 0.9% saline solution. Other chemicals were of analytical grade unless otherwise indicated. To analysis CisPt present in cells, exosomes, cell culture medium and tumour tissues the elemental Pt content was detected, using a monoelemental Pt standard solution (Spex CertiPrep, Metuchen, NJ, USA).

### Cytotoxicity Assays

The sensitivity to CisPt of the tumour cell lines (Me501, Me30966, MCF7 and SW480) was measured by the Trypan blue exclusion method. The cells were cultured in different culture medium pH (pH 7.4, UNB and pH 6.0), and were treated at different time points with 2.5, 5, 10, 20 and 40 µM of CisPt.

Cells were harvested by trypsinization. An aliquot of each cell line resuspended in phosphate buffered saline (PBS) was diluted 1∶1 (vol/vol) with 0.4% trypan blue. After 5 minutes incubation, cells were loaded onto a hemocytometer, and both live (unstained) and dead (blue-stained) cells were counted under a light microscope. Each treatment condition was tested at least in triplicate, and the mean value (% dead cells) was determined.

### Determination of Extracellular pH

The cells were collected by centrifugation (5 minutes at 500 g), and the cell culture supernatant was harvested for pH measurements. pH was determined using a Titroprocessor 726 pHmeter (Metrohm, Herisau, Switzerland) equipped with a glass microelectrode (LongLife; Metrohm).

### Exosomes purification from cell culture supernatants and plasma

Supernatants from human melanoma cell lines were harvested from 70–75% confluent cell cultures after 3 days in culture and isolated as previously described [32]. Briefly, after centrifugation of cells at 300 g for 10 minutes, supernatants were centrifuged at 1.200 g for 20 minutes followed by 10.000 g for 30 minutes. Supernatants were filtered using a 0.22 µm filter (Millipore Corp., Bedford, MA) and centrifuged at 100.000 g for 1 hour in a Beckman ultracentrifuge (Beckman Instruments Inc., Fullerton, CA, USA) in order to pellet the exosomes.

Experiments were performed with cells in exponential growth phase in acidic (pH 6.0–5.0), buffered (pH 7.4) and unbuffered media.

In order to obtain exosomes from plasma of CB.17 SCID/SCID mice engrafted with human melanoma, the blood was collected from mice ocular site under oxibuprocaina hydrochloride (Novartis Farma spa, Italy) anesthesia and was treated with EDTA. Subsequently, the exosomes were isolated as reported in a previous work [33].

### Determination of CisPt in cells, exosomes, cell culture medium and tumour tissue

In order to determine the CisPt content in all matrices, the Pt ion present in the drug is analyzed by means of a quadrupole based ICP mass spectrometer, Elan DRC II (Perkin-Elmer SCIEX, Norwalk, CT, USA). The instrumental settings and operative conditions are reported in the Table S1.

Prior to analysis, cells were lysed with a lysis buffer consisting of 150 mM NaCl, 20 mM Tris pH 7.4, 1% Nonidet P-40 and 10% glycerol, containing protease inhibitors (Hoffman-La Roche). The exosome pellets were lysed with 1% Triton X-100, 0,1 M Tris-HCl pH 7.4, 0.1% SDS and protease inhibitors (Sigma-Aldrich). Protein content was measured by Bradford assay (Biorad Laboratories, Hercules, CA, USA), according to the manufacturer's instructions.

Then, the cell and exosome lysates were digested by the addition of 200 µl or 50 µl of concentrated Super Pure Nitric Acid (Romil, Cambridge, Great Britain) respectively. They were kept at atmospheric pressure on a Mod Block heated plate (CPI international, The Netherlands) at 60°C for 2 hours. The final digested solutions were diluted with high purity deionized water (PBI International, Italy). Indium (1 µg/l) was added to specimens as internal standard, in order to correct the matrix effect and control the instrumental drift. The external standard calibration approach was chosen to quantify Pt by using the same matrix (lysing solution, nitric acid) as for the calibration standards. Finally, CisPt concentration in cells and exosomes has been expressed as ng of CisPt per mg of proteins present.

Cell culture medium samples were diluted 1∶100 with high purity water before the Pt analysis, adding only Indium as internal standard to minimize the effect of instrumental variation on the analytical signal. The level of the drug into the medium has been expressed as ng of CisPt per l of the solution.

To demonstrate the suitability of the analytical method the limit of quantification (LoQ) of Pt and the analytical variability were carried out. The LoQ is the lowest quantity of a substance that can be distinguished from the absence of that substance (a blank value) within a stated confidence limit. The limit of quantification is numerically equal to 10 times the standard deviation of the mean of blank determinations (n>20).

Moreover, to study the intra-cell line variability of cellular drug uptake, the following treatment was repeated 10 times, melanoma cells were incubated with 2 µM CisPt for 6 hours, collected and analyzed for the relative amount of proteins and CisPt (Fig.S1).

The CisPt chemical behaviour and its structure formula were investigated by a speciation analysis using a published method that combines High-performance liquid chromatography (HPLC) on-line with ICP-MS [34]. The HPLC system consisted of a Waters 600 binary pump (Waters, Milford, MA USA), a rheodyne injector filled with a 100 µl sample loop and a µbondpak C_18_ chromatography column (5 µ, 300 mm×3.9 mm) (Waters, Milford, MA USA). The aqueous mobile phase was filtered through a 0.45 µm membrane filter (Millipore, Molsheim, France), degassed by an ultrasonic bath before being pumped isocratically at a flow rate of 1.0 ml/min. At least 12 hours were required for equilibrating the column prior to each analytical run. The HPLC eluate was directly pumped into a cyclonic nebulizing chamber equipped with a Meinhard concentric nebulizer towards the ICP-MS torch. The operative instrumental conditions are reported in Table S2.

### *In vivo* tumour growth analyses

Female CB.17 SCID/SCID mice aged 4–5 weeks (Harlan; Correzzana, Milan, Italy) were kept under specific pathogen free conditions and fed *ad libitum*. The mice were housed in micro-isolator cages, and all food, water, and bedding were autoclaved prior to use. Mice were monitored for the duration of the *in vivo* experiments for body weight, hair ruffling, and the presence of diarrhea. All mice were killed by cervical dislocation at the end of the experiments, within two months after the injection of the human tumour cells (following the guidelines of the Istituto Superiore di Sanità/Italian National Institute of Health).

Each mouse of about 20 gr was injected subcutaneously in the right flank with 1×10^6^ Me30966 melanoma cells which were resuspended in 0.2 ml RPMI 1640. At least 5 mice were used for each treatment group, for a total of 10 mice/experiment.

Once tumours became evident, PPI was administered, 4 times per week, by intraperitoneal injection.

After about 6 weeks of PPI treatment, CisPt was administered intraperitoneally 2 times per week with a dose of 0,1 mg/mouse. The control group was treated with DMSO/saline solution.

Tumour growth was estimated 2 times per week with caliper by the following formula: tumour weight (mg) =  length (mm)×width^2^ (mm)/2, accordingly to Geran et al. [35].

### Ethics Statement

Animal experimentation is regulated in Italy by the *Legislative Decree* 116/92, which is the Italian enforcement of the European directive 86/609/EEC.

According to the above-mentioned *Legislative Decree* 116/92, protocols implying the use of laboratory animals for research purposes need to be approved by experts from ISS (National Institute of Health) and subsequently authorized by the Italian Ministry of Health.

The animals used in our experimentation were included in the research protocol “Comparison in *vivo* on efficacy of different proton pump inhibitors in cancer therapy; evaluation of their impact citotoxic in combination with chemotherapy drugs, and qualitative/quantitative analysis of human tumor exosomes” that was approved by the experts from ISS (**Service for Biotechnology and Animal Welfare**) and authorized by the **Italian Ministry of Health with the Decree n**° **DM 255/2012-B of 22/10/2012.**

### CisPt analysis in tumour tissue

The tumours were dried in an oven for 12 hours at 105°C and were completely digested by the addition of concentrated Super Pure Nitric Acid. The samples were treated with the same protocol used for cells and exosomes. Final results were expressed as mg of CisPt per g of tissue.

### ELISA for exosome detection

The ELISA test for the exosome detection (Exo-test, PCT/EE2009/000001) was performed as previously described [36]. Briefly, 96-well plates were coated with polyclonal anti-Rab-5b antibody (clone A-20, Santa Cruz) and incubated overnight at 4°C. After washes, exosomes purified from SCID mice-derived plasma were incubated overnight at 37°C. After washes, anti-CD63 mab (clone H5C6, Pharmingen Mississauga, ON) was incubated for 1 hour at 37°C. After the incubation with HRP-conjugated anti-mouse antibody, the results were analysed, recording the optical densities at 450 nm, by a microplate ELx800 reader (BioTek instruments, Vermont, USA).

### Statistical Analysis

Results are expressed as the means S.D. Paired Student's t tests and ANOVA one way, followed by a Bonferroni t-test, were used to examine group differences. *p*<0.05 was regarded as significant (*). Data are representative of at least three different experiments

## Results

### Analytical performance

The first set of experiments was performed to demonstrate the suitability of the analytical method used for the CisPt quantification in cellular and exosomes samples.

Table S3 reported the values of LoQs and intra-day precision regarding cells and exosomes. As for cells and exosomes, the LoQ was expressed as ng of CisPt per mg of protein (1×10^6^ cells  =  0.36 mg of protein). The maximum value for intra-day precision expressed as coefficient of variation in cells and exosomes digested solutions was 7.5%. This value is conceivable for a low level of CisPt.

A further set of experiments was aimed at evaluating the reliability and repeatability of our models, including the cells growing conditions and drug CisPt uptake. To this purpose, a parallel test on CisPt uptake of ten repeated Me30966 cell cultures was carried out and the variation coefficient was of 8.7% (Fig.S1). The cells were cultured at pH 7.4 for 3 days before being incubated with CisPt (final concentration 2 µM) for 6 hours. The CisPt content of the cells and the exosome released were measured and normalized to protein content.

Although the study was carried out in biological systems, the results obtained showed the suitability of the method in order to study the relationship between the level of CisPt in either the cells or exosome preparations and the pH of the culture medium. In fact, a variation of uptake higher than 9% could be accepted as significant and not due to the analytical inaccuracy.

### Cisplatin cellular resistance

**Figure 1:**
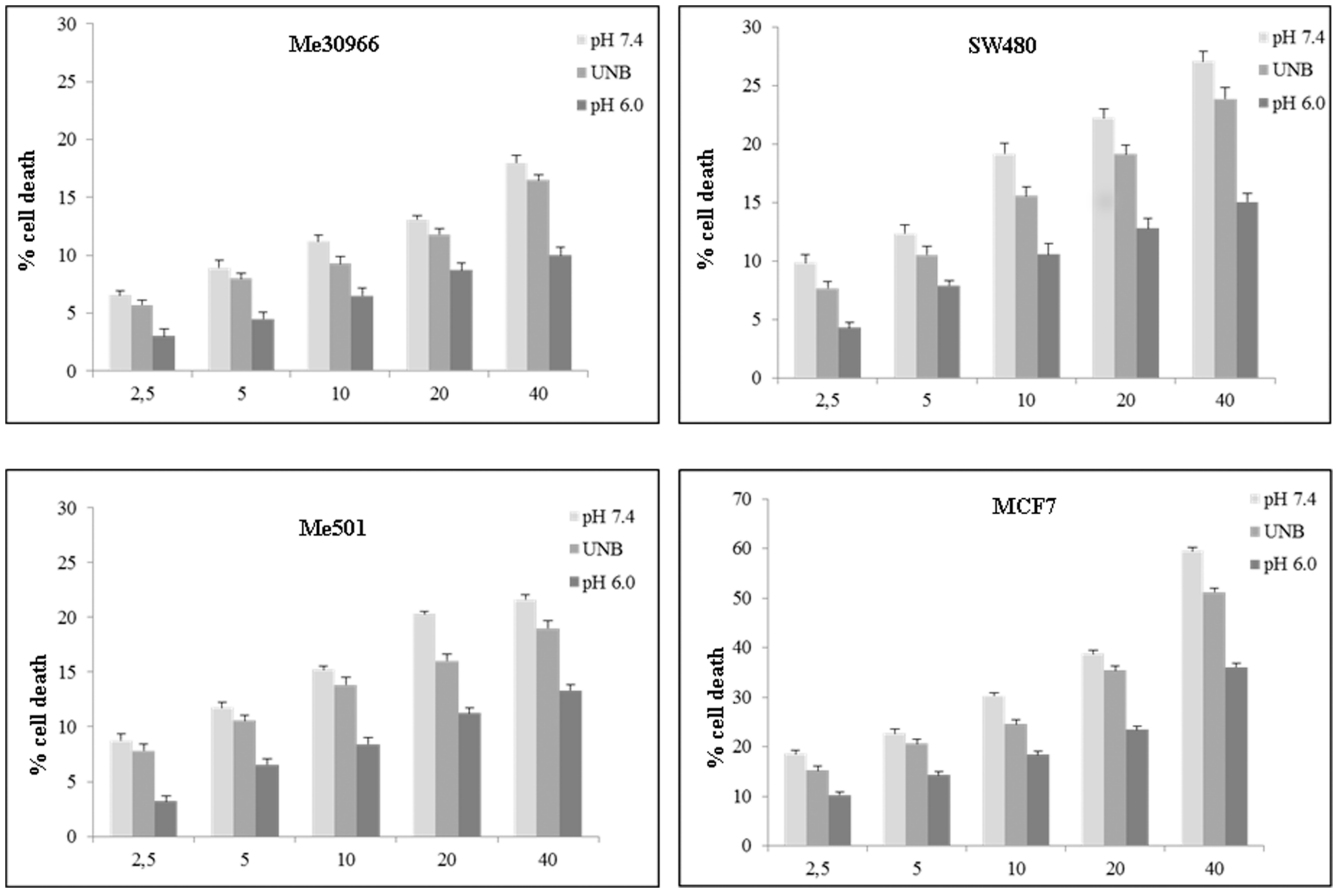
Cytotoxicity assay by Trypan blue exclusion method. Me30966, Me501, SW480 and MCF7 cell lines were incubated at pH µM of CisPt. Representative of three independent experiments are reported.

In a first set of experiments we analyzed the CisPt toxicity against different human tumour cell lines such as metastatic melanoma, breast cancer, colon carcinoma by the trypan blue exclusion method. To this purpose we performed a dose-response curve of human tumour cell lines cultured for two days at different pH conditions (pH 7.4, UNB and pH 6.0) and exposed to 2.5, 5, 10, 20 and 40 µM of CisPt. The results in Fig.1 showed that the tumour cell lines exhibited different sensitivity to the CisPt and that the acid culture condition reduced sensitivity to cisplatin in all tumour cell lines tested. We identified Me30966 metastatic melanoma cells as the most CisPt resistant cancer cell line while the MCF7 breast carcinoma was the most CisPt sensitive cell line (see the results of kinetic experiments, Fig.S2). In a separate set of experiments we confirmed that normal human cells, such as peripheral blood mononuclear cells (PBMC), showed a high cell death level in acidic conditions, (more than 60%, after 24 h of cellular incubation), as shown in Fig.S3, and therefore not useful for testing both CisPt effectiveness at different pH condition and not suitable to test the activity of PPI, that are pro-drugs needing low pH to be transformed into the active molecule.

### Role of extracellular pH in drug uptake

**Figure 2:**
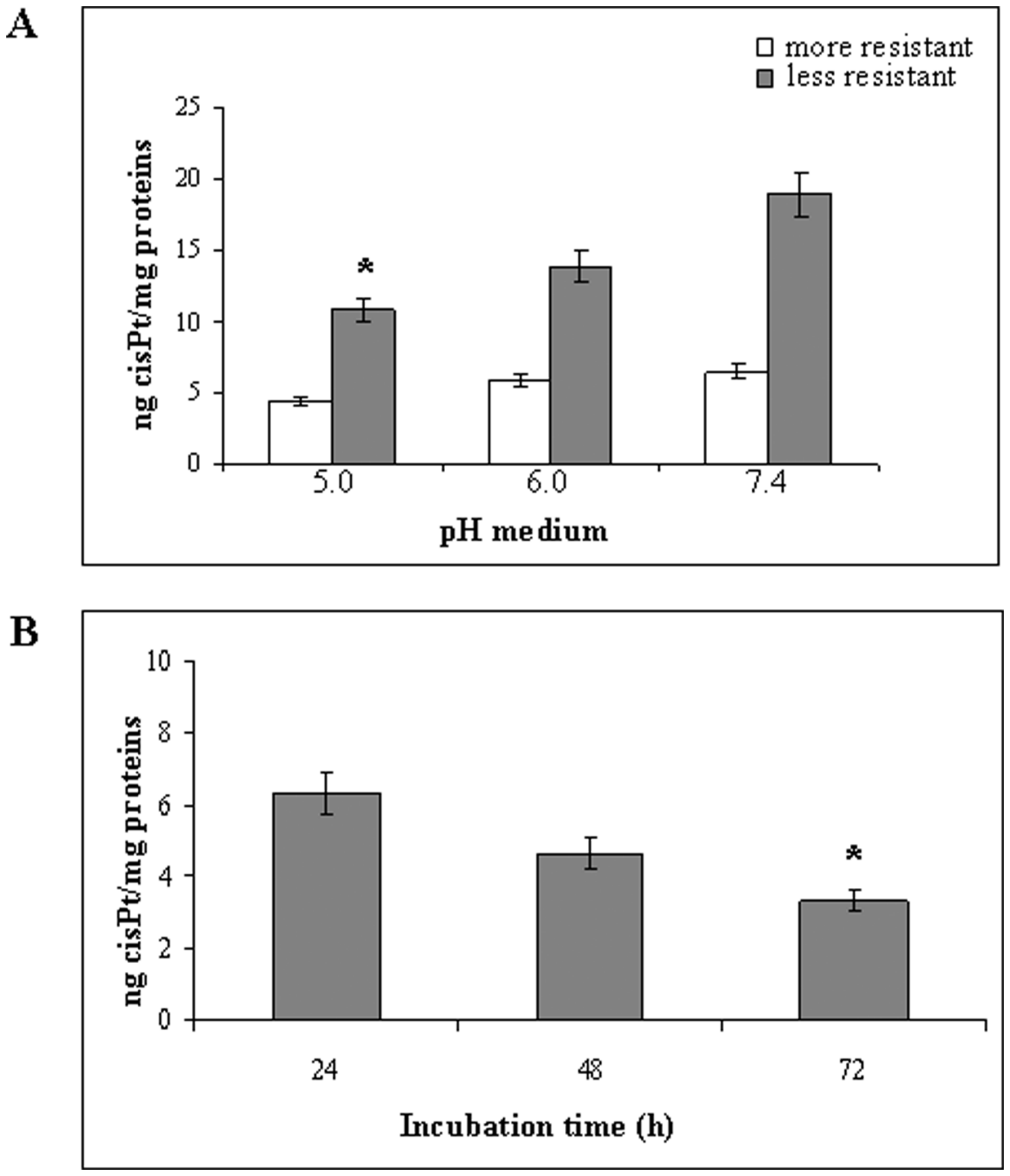
Analysis of intracellular CisPt at different pH. A: Intracellular CisPt level in more drug-resistant (Me30966) and less drug-resistant (MCF7) cells at different pH (5.0, 6.0 and 7.4) of culture medium. Significance (p<0.05) refers to CisPt level at pH 5.0 compared to pH 7.4. B: Intracellular CisPt level in Me30966 cells in function of different incubation times (24, 48, 72 hours) in UNB condition before drug administration. Significance (p<0.05) refers to CisPt uptake after 72 hours compared to 24 hours. Representative of three independent experiments are reported.

In the following experiments, we analyzed the effect of acidic pH on the CisPt uptake by human tumour cells. To this purpose the CisPt content by human tumour cell lines with different level of drug resistance (low: MCF7; high: Me30966) was measured at different pH conditions (pH 7.4, pH 6.0 and pH 5.0). Cell lines were cultured for two days with different pH culture media and then exposed to 2 µM CisPt for 6 hours. The CisPt uptake was measured after repeated washing in order to remove all free drug before analysis. The results showed that the acidic condition reduced the CisPt uptake by both cell types, while with different extents (Fig.2A).

Me30966 cells were next chosen for additional experiments on drug uptake as a function of culture medium pH, because these cells are more able to acidify the culture medium respect to the less resistant cells. In fact using an unbuffered medium (UNB) in order to allow a spontaneous culture medium acidification by tumour cells, Me30966 progressively acidified reaching at 72 hours incubation the lowest pH of 6.70 in respect to the pH of 6.84 of less resistant MCF7 cells.

Thus, we performed the following set of experiments by treating human melanoma cell cultures with 2 µM CisPt for 6 hours in UNB condition and evaluating the CisPt uptake at different time points. The results showed clearly that following spontaneous acidification, after 72 hours of incubation CisPt amount decreased to about 50% (Fig.2B), supporting the hypothesis that acidification of tumour cells microenvironment is a key factor in the melanoma resistance to cisplatin. Moreover, the decrease in CisPt cellular uptake was not due to reduced cell viability inasmuch as after 72 hours of incubation up to 95% of melanoma cells were viable (data not shown).

### A role of exosomes in melanoma resistance to cisplatin

Recent studies suggested that CisPt, once entered into tumour cells, may be sequestered into acidic vesicles belonging to a secretory pathway [28]. It might be therefore conceivable that exosomes, representing key actors of the cell vesicle-mediated secretory pathway, could participate to this pathway of cellular drug elimination, including cisplatin as well. To investigate this hypothesis we analysed the CisPt content of exosomes released by tumour cells grown at various pH conditions. The results showed that exosomes released by cultured resistant melanoma cells, previously treated with a fixed dose of CisPt, contained various amounts of the drug depending on the pH conditions of the culture medium. In fact, the level of CisPt in the exosomes was higher in both acidic pH (pH 6.0 and pH 5.0) than at pH 7.4 (Table 1). This result was consistent with a previous evidence from our group showing that acidic pH increased exosome release by tumour cells [31], thus probably favouring the CisPt elimination through the exosome pathway.

**Table 1:** Content of CisPt in the exosomes.

pH medium	ng CisPt
UNB	0.59±0.14
pH 7.4	0.52±0.13
pH 6.0	0.70±0.18
pH 5.0	0.87±0.15 *

Content of CisPt in the exosomes per mg of total proteins at different pH. Data are representative of three experiments. *p<0.05.

### Effect of extracellular microenvironmental buffering via PPI pre-treatment on uptake and exosome-mediated elimination of CisPt

**Figure 3:**
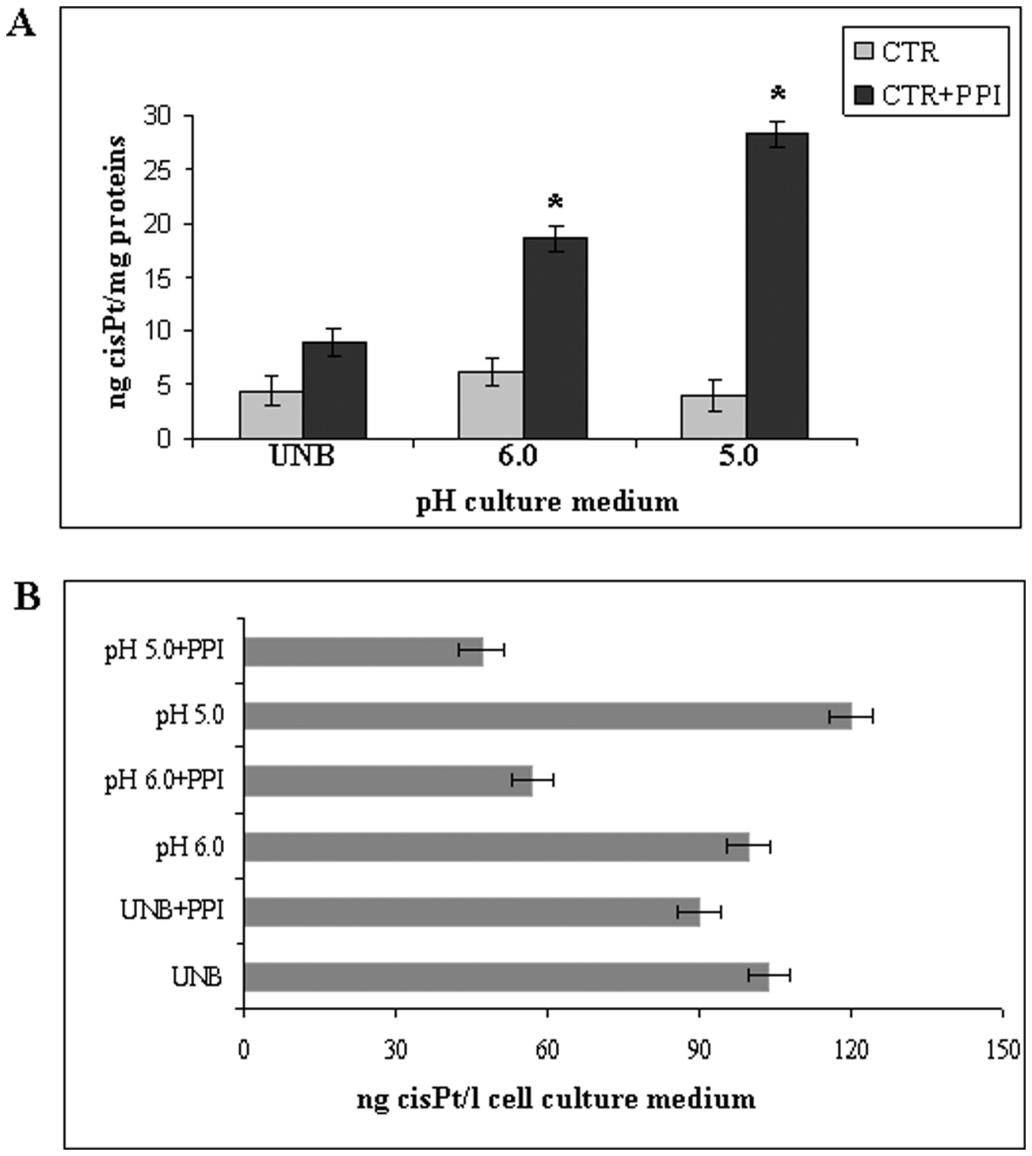
Effect of PPI on CisPt cellular uptake. A: Effect of PPI on CisPt uptake in Me30966 cells in function of different pH (UNB, 6.0 and 5.0) culture medium. CTR: Me30966 cells incubated with CisPt, without PPI pre-treatment; CTR+PPI: Me30966 cells pretreated with PPI and then incubated with CisPt. Significance (p<0.05) refers to CisPt cellular uptake at 5.0 and 6.0 pH in comparing PPI pretreatment to CTR in UNB medium. B: Effect of PPI on drug release at different pH (UNB, 6.0 and 5.0). CisPt ng/l present in cell culture medium obtained from cells pretreated with PPI and then incubated with CisPt. p<0.05. Representative of three independent experiments are reported.

Our previous studies have shown that treatment of either tumour cells or tumours with proton pump inhibitors (PPI) induced both chemosensitization [23] and impairment of exosome release by tumour cells [31]. We further showed that this effect was due to a clear anti-acidic effect of PPI at both cellular and tumour levels [14], [23], [37]. We thus explored the hypothesis that acidic extracellular pH might have a role in both inhibiting CisPt entry into the cells and favouring CisPt-containing exosome release, by treating human melanoma cells with PPI and evaluating CisPt cellular/exosomes content as compared to the CisPt concentration in cell culture supernatants. First, we compared the effect of PPI on drug uptake and drug elimination at different pH (pH 5.0 and 6.0) and in UNB conditions. The treatment schedule we used was one day pre-treatment with fixed dose of PPI (50 µM Lansoprazole) followed by 6 hours incubation with 2 µM CisPt, then analyzing the CisPt amount in cells, exosomes and cell culture medium. The results showed that PPI pre-treatment increased the CisPt cellular uptake as compared to untreated cells in an acidic-depend manner (Fig.3A), supporting the importance of acidity in allowing a full activation of PPI, as suggested by previous finding [14], [23], [25]. These data was supported by the decreased amount of CisPt in supernatants of melanoma cell culture receiving PPI, again depending on the *a priori* cell culture pH condition (Fig.3B).

Finally, melanoma cells pre-treatment with PPI at low pH condition induced a 50% reduction in the CisPt content in the exosome population purified from the cell culture supernatant, as compared to the exosome purified from supernatant of cell cultures that did not treated PPI (Table 2). These data support the hypothesis that PPI pre-treatment at the same time may lead to both exosome release inhibition and an increased drug retention by tumour cells.

**Table 2:** Content of CisPt in the exosomes from +/− PPI pre-treatment.

	ng CisPt	
pH medium	CisPt	CisPt+PPI
UNB	0.87±0.32	0.64±0.17
pH 6.0	1.69±0.31	0.71±0.11 *
pH 5.0	1.83±0.30	0.84±0.16 *

Content of CisPt in the exosomes per mg of total proteins at different pH with/without PPI pre-treatment. Data are representative of three experiments. *p<0.05.

### The chemical activation status of CisPt in exosomes and tumour cells

The antitumour activity of platinum compounds is related to a set of structure-activity relationships. Activity against tumours could be expressed by this general formula: *cis-*[CisPtX2(Am2)2], where X is the leaving group (Cl^−^ in CisPt) and Am is an inert amine with at least one N-H moiety.

**Figure 4:**
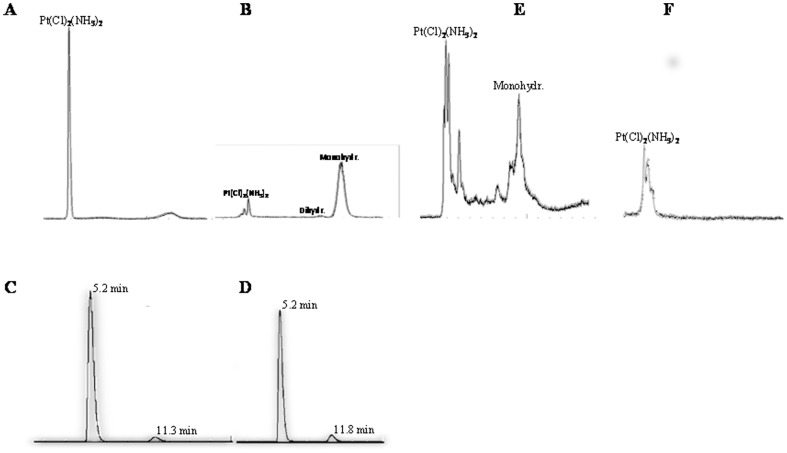
HPLC-Q-ICP-MS chromatograms of a standard solution of CisPt. Chromatogram of CisPt solution dissolved in NaCl 0.9% (A) and in water after sonication (30 min) at 80°C (90 min) (B). Chromatograms of CisPt dissolved in cell culture medium after dissolution (C) and after 6 hours incubation (D), peak of native form of Cis-Pt at 5.2 min; peak of monohydrated CisPt at 11.3 min. Chromatogram of Me30966 cells lysate solution containing native and monohydrated forms of CisPt (E); chromatogram of exosomes lysate solution containing only the native form of the drug (F). Representative of three independent experiments are reported.

In the presence of a fluid media containing high concentration of chloride (NaCl 0.9%) the drug remains in its native form (Fig.4A). Otherwise, in fluids with low Cl^−^ concentration both monohydrated complex *cis-*[CisPtCl(NH3)2H2O]^+^ and, to a lesser extent dihydrated complex *cis-*[CisPt(NH3)2(H2O)2]^+^ is formed (Fig.4B).

In the above mentioned media, the CisPt can ionize into positively charged protonated species which exists in equilibrium with uncharged, unprotonated forms of the drug. The uncharged form of ionizable drug typically crosses the plasma membrane of cells fairly readily, this being a requirement for an effective drug activity. Both uncharged and charged protonated species of CisPt were identified and separated in the culture media by means of HPLC-Q-ICP-MS. The chromatographic separation was carried out also in the cell and exosome lysate.

Samples were taken 5 minutes after the dissolution of the drug into the medium (time 0) and at the end of the incubation period of 6 hours (time 1). In figure 4 the chromatographic separation of CisPt forms at time 0 (C) and time 1 (D) is reported. The peak with a retention time (RT) of about 5 minutes represents the native form of the drug, while, the monohydrated complex shows an eluting peak at about 11 min. After a period of time of 6 hours, only a slight increase of the peak of hydrated form (RT 11 min) can be observed. Therefore, most of the drug, during the incubation time, remained in its native uncharged unprotonated form, which is able to cross the cell membrane. Figure 4 reported the chromatographic pictures of the drug found into either cells (E) or exosomes (F). CisPt was measurable in the cytosol of the cells where the Cl^−^ concentration is about ten times lower than in the extracellular fluids, mostly in its monohydrate therapeutically active form, supporting previous reports [38], [39], while the CisPt measured in the exosome preparations was essentially in its native form.

### Effects of PPI on CisPt tumour uptake in human tumours/SCID mice xenografts

**Figure 5:**
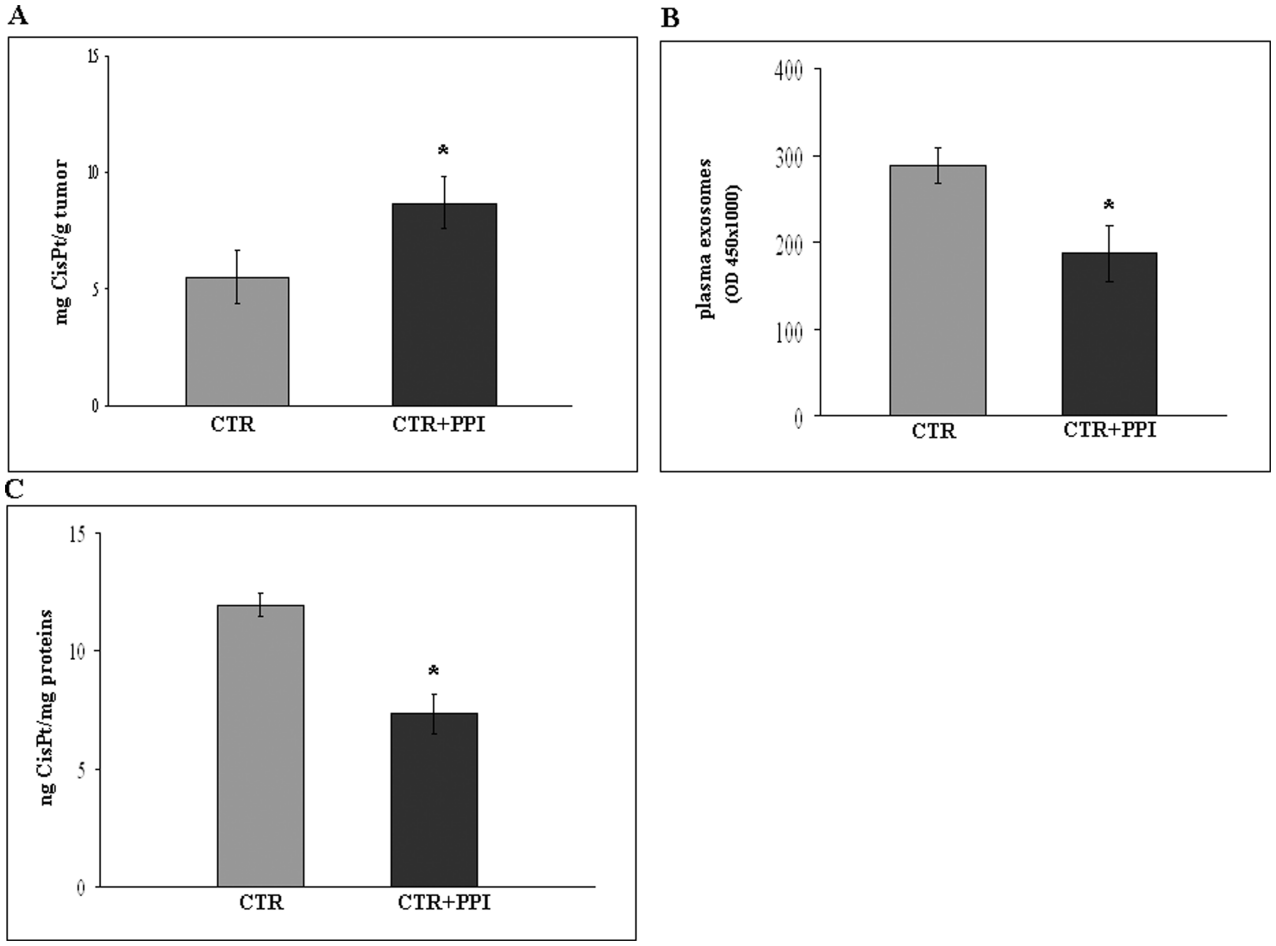
*In vivo* effect of PPI on CisPt uptake. A: Effect of PPI (25 mg/kg) on CisPt uptake by human tumour in SCID mice model. CTR: SCID mice treated with CisPt (0,1 mg/mouse), CTR+PPI: SCID mice pretreated with PPI and then with CisPt. p<0.05. B: Effect of PPI in SCID mice plasma on exosomes tumour release analysed by Exo Test. p<0.05. C: Effect of PPI on CisPt content in exosomes circulating in SCID mice. CTR: plasma exosomes of SCID mice treated with CisPt, CTR+PPI: plasma exosomes of SCID mice pretreated with PPI and then with CisPt. p<0.05. Representative of three independent experiments are reported.

To assess the potential *in vivo* relevance of the *in vitro* results, we performed a set of experiments in a human/mouse model system [40] represented by CB.17 SCID/SCID mice injected subcutaneously with human melanoma cells. In particular, CB.17 SCID/SCID mice engrafted with human tumour cells were pre-treated with a fixed dose of Lansoprazole (12,5 mg/kg) for 3 consecutive days/week, previously shown to be highly effective against melanoma [14]. Six weeks later, animals were treated with 0,1 mg of CisPt once a week for 2 weeks. The results showed that human tumours obtained from xenografts pretreated with PPI contained more CisPt as compared to tumours from xenografts treated with CisPt only (Fig.5A), even if the tumours analysed did not show differences in weight (data not shown).

Moreover, to test whether circulating human tumour-derived exosomes could contain CisPt, we purified exosomes from plasma of single xenograft (as described in Materials and Methods) 6 weeks after the engraftment with human melanoma cells, thus analyzing the CisPt content into the plasmatic exosome purifications from xenografts treated with either CisPt alone or PPI/CisPt combination. The results showed that on one hand PPI induced in the xenografts a marked reduction in the level of plasmatic exosomes (Fig.5B), on the other hand PPI induced a significant reduction of the CisPt content in plasmatic exosomes with respect to the control (Fig.5C).

## Discussion

Melanoma is by far one of the most chemoresistant malignant tumours, showing an intrinsic resistance to Cisplatin as well. Between the mechanisms shown to have a role in resistance of cancer cells to Cisplatin there are increased efflux, or increased inactivation by sulfhydryl molecules, such as glutathione; altered expression of proteins in signal transduction pathways that control apoptosis; increased DNA repair [41]. However, while without a clear molecular targetting, microenvironmental low pH appears to exert a major role in resistance to chemotherapy, proliferation and metastatic behavior of malignant tumours [15], [18], [42]. In fact, the extracellular pH of normal tissue is neutral, the interstitial pH of tumour is acidic and the tumour cells have developed the capacity of surviving in hypoxic-acidic environment, condition not permissive to the normal cells. This selective advantage is exploited by the tumour cells to markedly impair the uptake of weakly basic chemotherapeutic drugs and as a consequence their effect on tumours. All in all, tumour acidity does not inhibit intracellular mechanism/s related to the effectiveness of the drugs, but it hampers their entry within a cell, thus getting under ways a simple, rough but extremely efficient mechanism that makes real poisons unable to operate. However, in this study we show that the same cells use an additional mechanism of resistance, that is the elimination of chemotherapeutics through extracellularly released nanovesicles, called exosomes. The two phenomena are linked in a complementary way, inasmuch as low pH increases the exosome release by tumour cells. In a previous study [23], we have extensively investigated the level of pH dependent resistance of various human tumour cell lines against different chemotherapeutics, including CisPt. In this study we wanted to obtain more mechanistic insights of CisPt tumour resistance in extreme experimental settings, such as the ability to grow in very acidic condition and high level of exosome release. In fact, this study has shown that in melanoma cells cultured at different low pH conditions (i.e. 6.0 and 5.0), there was an impaired CisPt uptake by tumour cells as compared to melanoma cells cultured at the physiological pH of 7.4. We further confirmed this result culturing melanoma cells in unbuffered condition, leading to a marked lowering of the medium pH (round 0.4 units, data not shown) [23]. In this more “natural” acidic condition we had again a reduced CisPt uptake in melanoma cells, further supporting a clear role of the acidic microenvironment in chemoresistance.

However, among the several mechanisms involved in the phenomenon of drug resistance, including decreased uptake or neutralization of weakly basic drugs by the acidic tumour microenvironment, there is the sequestration of chemotherapeutic drugs within lysosomal vesicles [15], [17], [26]. In this study we have also provided evidence that exosomes have a role in chemoresistance by eliminating chemotherapeutic drugs (CisPt) into the extracellular microenvironment. In fact, the results showed that exosomes purified from supernatants of melanoma cells treated with CisPt contained detectable levels of the drug and that the exosome levels of CisPt was higher in acidic than in buffered conditions (Table 1). Moreover, HPLC analysis has shown that CisPt content in the exosome samples was in its native uncharged/unprotonated form, that is the molecule in its unmodified chemical form. An hypothesis might be that the exosomes incorporate the drug immediately after the cell uptake or anyway before a significant activation by hydration occurred. Once incorporated into exosomes, the drug remains in its native unhydrated form, probably because the Cl^−^ concentration within exosomes is similar to extracellular fluids. However, independently from the precise mechanism/s underlying this phenomenon, we provide evidence supporting that once the drug entered into cells, it was rapidly trapped by exosomes, thus preventing CisPt activation mechanism and targetting to the specific cell compartments, in turn contributing to the framework leading tumour cells to free themselves from the cytotoxic drug. Intriguingly, the exosome release in acidic condition was higher than in the 7.4 physiological pH and the acidic exosomes contained a greater amount of CisPt, providing an evidence that the exosome-mediated CisPt elimination may be highly operating within the tumour mass that is intrinsically acidic [12], [14].

Our results are supported by previous work showing that the majority of the intracellular fluorescent form of Cisplatin was associated with vesicular structures and that the fluorescent Cisplatin instead of diffusing freely through the cytoplasm, is sequestered into specific vesicles presumably by membrane-bound proteins or other unknown mechanisms [26], [28], [43].

Proton pump inhibitors (PPI) may revert chemoresistance and increase chemosensitivity of different human tumour cells [23], [44]. PPI pre-treatment was associated with the inhibition of V-H(+)-ATPase activity and the increase of both extracellular pH and the pH of lysosomal organelles is consistent with a cytoplasmic retention of the cytotoxic drugs [23]. PPIs are protonable weak bases which selectively accumulate in acidic spaces and need an acidic pH to be transformed in the active molecule [45]. This is a key property of PPI as proton pump inhibitors, inasmuch as other molecules that directly inhibit V-ATPase, such as bafilomycin, are extremely toxic for normal cells This is because V-ATPase are ubiquitous proton pumps, whose function is key for many organs and compartments of the human body. So, PPI, for their acidic-dependent activation, are molecules that avoid V-ATPases inhibition-derived toxicity against normal cells.

In this study, we used the unbuffered culture conditions (UNB) as a model of spontaneous microenvironmental acidification by tumour cells, in order to allow a more “natural” activation of PPI. We found that pre-treatment with PPI induced the doubling of the CisPt concentration within the cells cultured in UNB condition and approximately 5 times more than the CisPt intracellular content in acidic condition as compared to untreated controls. Notably, the same acidic condition that are responsible to the weak bases neutralization is the most suitable for the full activation of PPI. All in all while protonation, by H^+^, for the vast majority of drugs induces inactivation of the drug, through extracellular protonation, the same condition induces the “full activation” of PPI.

These results were confirmed in experiments *in vivo*, using a human/mouse model system represented by CB.17 SCID/SCID mice injected subcutaneously with human melanoma cells. The results showed that following PPI pre-treatment, human tumours in mice contained more CisPt as compared to the control xenografts, while not showing significant differences in term of weight, due to the very tight time points used to have a reliable CisPt quantification. Interestingly, PPI pre-treatment induced at the same time a marked inhibition of the tumour exosome spill-over into the blood stream and a substantial reduction of CisPt in the plasmatic exosomes, as compared to the exosomes purified from the plasma of xenografts treated with CisPt alone. These data, support the evidence that exosomes are stable vesicular structures able to circulate in different biological fluids [36], [46] and that the PPI pre-treatment is able to alter the uptake of chemotherapeutics [23].

A previous report from our group based on an *in-vitro* pharmacokinetic study [47], demonstrated that following Cisplatin incubation at 37°C with human plasma, the 80% of the drug after only 2 hours of incubation was bound to albumin and globulin as well as to unidentified protein species of relatively low molecular weight. In fact, the released CisPt may be either free drug or a conjugate/complex with cellular proteins to which it has become bound [28]. After 2 and 4 hours of incubation, the unbound aliquot of the drug (named “free Cisplatin”), that was the therapeutically active form, was 20% and 10% of the total drug added, respectively. Moreover, after 24 h the free CisPt completely disappeared. This study adds much to the knowledge on the pharmacokinetic of CisPt providing the evidence for a role of exosomes in the extracellular elimination of this molecule and suggesting a specific function of exosomes in the routing of intracellular drug.

Our results provide clear evidence that human malignant cells may reduce effectiveness of potent anti-cancer drugs through contemporary two mechanisms, that are extracellular acidification and release through nanovesicles called exosomes. Moreover, this study shows that PPI pre-treatment really increases the uptake of anticancer drugs, such as Cisplatin, through both reduction of extracellular pH and inhibition of tumour exosome release. PPI may represent a model of future anticancer molecules using tumour acidity as a target [48].

## Supporting Information

Figure S1 **CisPt uptake in 10 repeated experiments of Me30966 cell culture.**
(TIF)

Figure S2 **Cytotoxicity assay by Trypan blue exclusion method. MCF7 and Me30966 cells were treated with CisPt for 24, 48 and 72 hours.**
(TIF)

Figure S3 **Cytotoxicity assay by Trypan blue exclusion method. PBMC resting were incubated for 24 and 48 h at pH 7.4, UNB and pH 6.0 conditions.**
(TIF)

Table S1 **Instrument settings and data acquisition parameters for Q-ICP-MS.**
(DOC)

Table S2 **HPLC-Q-ICP-MS operative conditions of the method.**
(DOC)

Table S3 **Limits of quantification (LoQs) of CisPt and intra-day precision (CV %) in cells and exosomes.**
(DOC)
