# Supplementary figures and images for: Exosome Release and Low pH Belong to a Framework of Resistance of Human Melanoma Cells to Cisplatin
Source: PLoS One. 2014 Feb 6;9(2):e88193. doi: 10.1371/journal.pone.0088193 (PMC3916404; doi:10.1371/journal.pone.0088193)

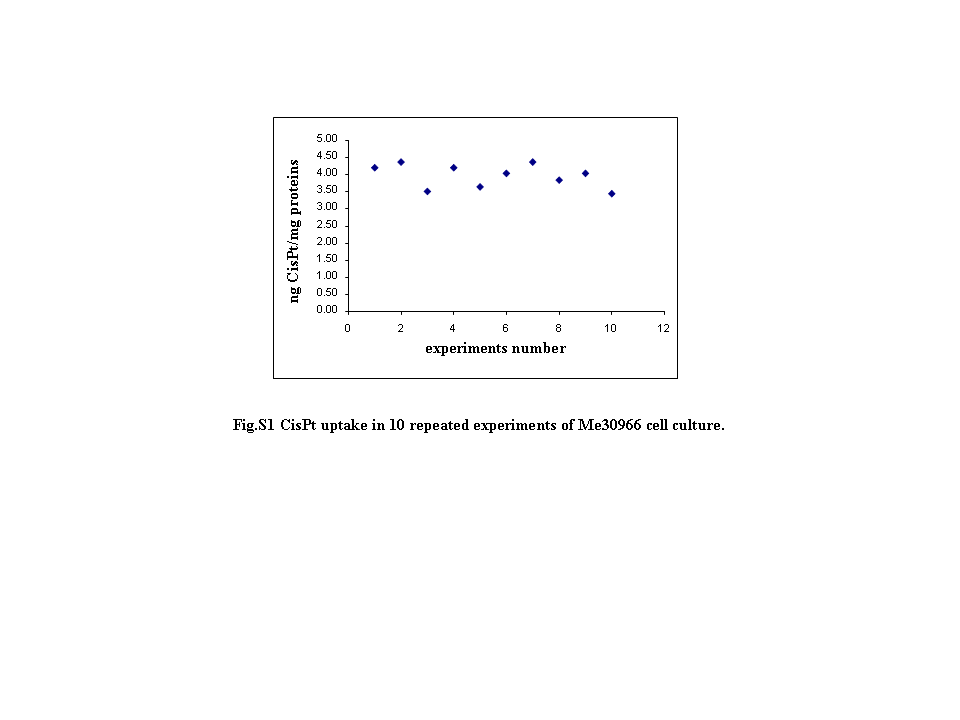

Supplement: Figure S1 — CisPt uptake in 10 repeated experiments of Me30966 cell culture. (TIF) [file pone.0088193.s001.tif]

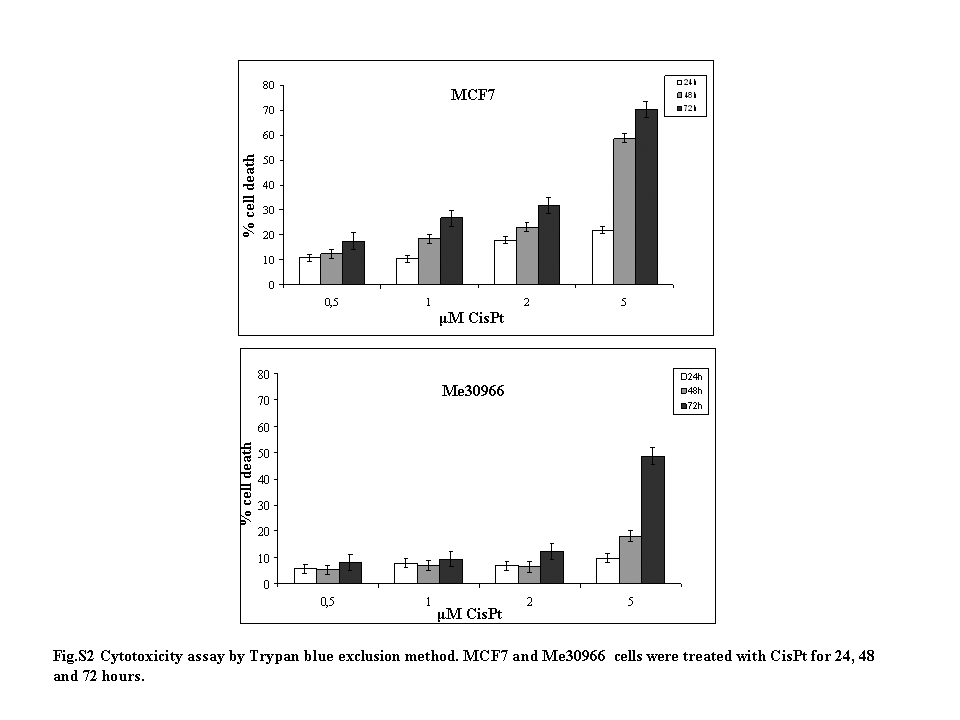

Supplement: Figure S2 — Cytotoxicity assay by Trypan blue exclusion method. MCF7 and Me30966 cells were treated with CisPt for 24, 48 and 72 hours. (TIF) [file pone.0088193.s002.tif]

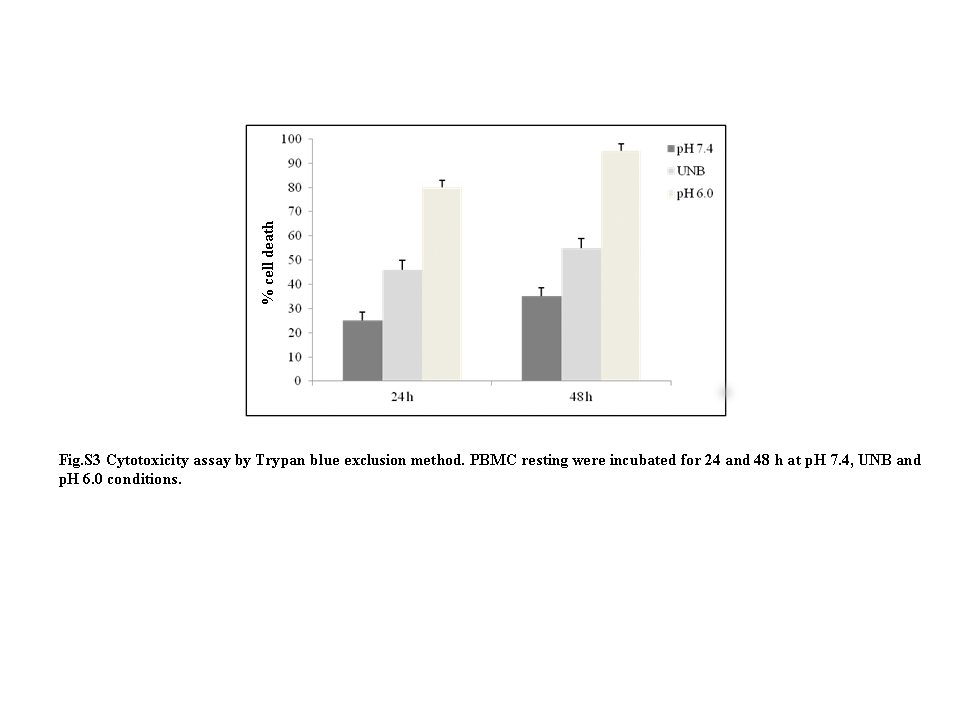

Supplement: Figure S3 — Cytotoxicity assay by Trypan blue exclusion method. PBMC resting were incubated for 24 and 48 h at pH 7.4, UNB and pH 6.0 conditions. (TIF) [file pone.0088193.s003.tif]
