# Supplementary material for: Exosome Release and Low pH Belong to a Framework of Resistance of Human Melanoma Cells to Cisplatin
Source: PLoS One. 2014 Feb 6;9(2):e88193. doi: 10.1371/journal.pone.0088193 (PMC3916404; doi:10.1371/journal.pone.0088193)
Supplement: Table S1 — Instrument settings and data acquisition parameters for Q-ICP-MS. (DOC) [file pone.0088193.s004.doc]

**Table S1. Instrument settings and data acquisition parameters for Q-ICP-MS**

Instrumentation Elan DRC II (Perkin Elmer SCIEX, Norwalk, CT, USA)

Sample ucisPtake rate 400 µl min^-1^

Sample introduction Apex IR (ESI, Elemental Scientific Inc., Omaha, NE, USA): condenser, -5 ºC, heater 140 ºC

RF power 1400 W

Gas flow rates (l min^-1^) Plasma, 15; Auxiliary, 1.0; Nebulizer, 0.8

Interface CisPt cones

Extraction lens voltage OcisPtimized for maximum I (^56^Fe)

Sensitivity 400.000 c/s 1 ng/ml In

Internal standard ^115^In

Analytical Masses ^195^CisPt

Scanning mode Peak hopping

Dwell time (ms) 100

Number of replicate 8
