# Supplementary material for: Exosome Release and Low pH Belong to a Framework of Resistance of Human Melanoma Cells to Cisplatin
Source: PLoS One. 2014 Feb 6;9(2):e88193. doi: 10.1371/journal.pone.0088193 (PMC3916404; doi:10.1371/journal.pone.0088193)
Supplement: Table S2 — HPLC-Q-ICP-MS operative conditions of the method. (DOC) [file pone.0088193.s005.doc]

**Table S2. HPLC-Q-ICP-MS operative conditions of the method**

**HPLC**

Instrumentation Waters 600 binary pump

Stationary phase Waters *µ*bondpak C_18_ (5µ, 300mm X 3.9 mm)

Mobile phase 3% Methanol 0.15 mM SDS pH 2.5 triflic acid

Gradient Isocratic

Injection volume 50 µl

**ICP-MS**

RF power 1400 W

Gas flow rates (l min^-1^) Plasma, 15; Auxiliary, 1.0; Nebulizer, 0.8

Sample introduction Concentric Meinhard nebulizer with cyclonic spray chamber

Analytical Masses ^195^CisPt

Sensitivity 400.000 c/s 1 ng/ml In

Scanning mode Transient signal

Dwell time (ms) 100
