# Supplementary material for: Exosome Release and Low pH Belong to a Framework of Resistance of Human Melanoma Cells to Cisplatin
Source: PLoS One. 2014 Feb 6;9(2):e88193. doi: 10.1371/journal.pone.0088193 (PMC3916404; doi:10.1371/journal.pone.0088193)
Supplement: Table S3 — Limits of quantification (LoQs) of CisPt and intra-day precision (CV %) in cells and exosomes. (DOC) [file pone.0088193.s006.doc]

**Table S3. Limits of quantification (LoQs) of CisPt and intra-day precision (CV %) in cells and exosomes**

Cells Exosomes

LoQ (ng CisPt/mg proteins) 0.001 0.001

CV (%) 5.5 7.5
